# Psychometric Properties of the Perinatal Anxiety Screening Scale Administered to Italian Women in the Perinatal Period

**DOI:** 10.3389/fpsyt.2021.684579

**Published:** 2021-06-22

**Authors:** Alexia Koukopoulos, Cristina Mazza, Lavinia De Chiara, Gabriele Sani, Alessio Simonetti, Georgios D. Kotzalidis, Giulia Armani, Gemma Callovini, Marco Bonito, Giovanna Parmigiani, Stefano Ferracuti, Susanne Somerville, Paolo Roma, Gloria Angeletti

**Affiliations:** ^1^Department of Human Neuroscience, Sapienza University of Rome, Rome, Italy; ^2^Lucio Bini Centre, Rome, Italy; ^3^Department of Neuroscience, Imaging and Clinical Sciences G. d'Annunzio University of Chieti-Pescara, Chieti, Italy; ^4^Department of Neurosciences, Mental Health, and Sensory Organs (NESMOS), Sapienza University of Rome, Faculty of Medicine and Psychology, Sant'Andrea University Hospital, Rome, Italy; ^5^Institute of Psychiatry, Università Cattolica del Sacro Cuore, Rome, Italy; ^6^Department of Psychiatry, Istituto di Ricovero e Cura a Carattere Scientifico, Fondazione Policlinico Universitario Agostino Gemelli IRCCS, Rome, Italy; ^7^Menninger Department of Psychiatry and Behavioral Sciences, Baylor College of Medicine, Houston, TX, United States; ^8^APC Associazione di Psicologia Cognitiva, Rome, Italy; ^9^Department of Mental Health, Psychiatric Service of Diagnosis and Treatment, “San Camillo de Lellis” National Health System Hospital, Rieti, Italy; ^10^Dipartimento Materno Infantile, San Pietro Fatebenefratelli Hospital, Rome, Italy; ^11^Department of Psychological Medicine, King Edward Memorial Hospital, Subiaco, WA, Australia

**Keywords:** pregnancy, screening, psychometric properties, factor analysis, anxiety, perinatal

## Abstract

Literature stressed the importance of using valid, reliable measures to assess anxiety in the perinatal period, like the self-rated Perinatal Anxiety Screening Scale (PASS). We aimed to examine the psychometric properties of the Italian PASS version in a sample of Italian women undergoing mental health screening during their third trimester of pregnancy and its diagnostic accuracy in a control perinatal sample of psychiatric outpatients. Sample comprised 289 women aged 33.17 ± 5.08, range 19–46 years, undergoing fetal monitoring during their third trimester of pregnancy, with 49 of them retested 6 months postpartum. Controls were 60 antenatal or postnatal psychiatric outpatients aged 35.71 ± 5.02, range 22–50 years. Groups were assessed through identical self- and clinician-rating scales. Confirmatory Factor Analysis (CFA), Principal Component Analysis (PCA), Pearson's correlations and receiver operating characteristic were conducted for PASS. PCA and CPA confirmed four-factor structure with slight differences from the original version. Construct validity and test-retest reliability were supported. Cut-off was 26. The PASS correlated with principal anxiety scales. Despite small sample size, findings confirm reliability and validity of the Italian PASS version in assessing anxiety symptoms in the perinatal period. Its incorporation in perinatal care will improve future mother and child psychological health.

## Introduction

Studies on perinatal psychiatric disorders have focused for several years on depressive and psychotic symptoms (1, 2), with less attention paid to perinatal anxiety, probably due to the overlap of anxiety symptoms on depressive syndromes (3, 4). However, anxiety may be more common than depression among women during the first perinatal period (5–7) and significantly higher in the maternal population than in the general adult population (8). This could be due to the higher prevalence of medical conditions during pregnancy, that predispose to anxiety disorders (9). Furthermore, perinatal anxiety is associated with obstetric problems and can negatively affect offspring's emotional and cognitive development (10–19), possibly due also to connectivity changes shown in 32-week fetal brain (20). Taken together, these data suggest that anxiety in the perinatal period should be considered on its own.

Available data suggest that 13–25% of pregnant women (21, 22) and 11–21% of postpartum women (23) experience clinically significant anxiety, with an estimated 20.7% for having at least one anxiety disorder in the peripartum (95% highest density interval from 16.7 to 25.4%) (24). When diagnostic interviews were employed, the prevalence rate for any anxiety disorder during the first trimester was 18%, decreasing marginally to approximately 15% in the final two trimesters of pregnancy, with a continuous decrease postnatal pattern across the 1st year (8). A meta-analysis estimated the prevalence of anxiety disorders in the postpartum to be around 8.5% (25), compared to 10–15% of depression (26). A large percentage of perinatal women who do not meet diagnostic criteria for a specific anxiety disorder, experience clinically significant anxiety symptoms, which they distinguish from anxiety experienced at other times of life (27, 28). Pregnant women often have concerns about the health and well-being of their babies, labor and delivery, finances, and the maternal role and responsibilities. These concerns are termed pregnancy-related anxiety (PrA) or pregnancy-specific anxiety (29). Despite its demonstrated importance, pregnancy-specific anxiety is a relatively new concept in maternal and child health research (30). Clinically relevant symptoms of pregnancy-specific anxiety will differ from common worries and concerns by virtue of their intensity, persistence, and impact on a woman's functioning (31).

Perinatal anxiety has typically been measured by standardized anxiety scales. Controversy exists over which screening tools should be used during the perinatal period to detect perinatal anxiety and the cutoffs that should be adopted for identifying women at risk. General anxiety measures may include questions about physical symptoms of anxiety common in pregnancy. Hence it has been suggested that PrA needs its own scale, as it appears to have distinctive features that are not captured by the standardized anxiety scales (32).

In recent years, maternal mental health investigators have been trying to improve knowledge of perinatal anxiety and to develop screening tools for identification of specific symptoms. A good understanding of pregnancy related anxiety is of key importance so to carry out preventive interventions or treatment strategies. The American Congress of Obstetricians and Gynecologists (ACOG) and the US Preventive Services Task Force (USPSTF) widely recommend routine screening for anxiety at least once during the perinatal period with a standardized, validated tool (33, 34).

In the search for specific anxiety rating scales in the perinatal period, two scales were developed, the Postpartum Worry Scale (35) and the Postpartum Specific Anxiety Scale (36); however, these have not been validated for the entire perinatal period. The Perinatal Anxiety Screening Scale [PASS; (37, 38)] is a self-rating scale used to screen for anxiety disorders in the entire perinatal period. It has a four-factor structure, i.e., (1) acute anxiety and adjustment, (2) general worry and specific fears, (3) perfectionism, control, and trauma and (4) social anxiety.

Given the importance to assess anxiety symptoms specifically during the perinatal period, the main aim of the present cross-sectional/longitudinal study was to validate the psychometric properties of the self-rated PASS in a sample of Italian women undergoing mental health screening during their third trimester of pregnancy, and in those agreeing to be retested, 6 months postpartum. The second aim was to investigate its diagnostic accuracy in a clinical perinatal sample and compare it with the HAM-A, a gold standard clinician-rated instrument.

## Materials and Methods

### Participants

The study involved two separate groups of participants. Demographic data of both samples are shown in Table 1. Participants provided written informed consent, in accordance with all applicable regulatory and Good Clinical Practice guidelines and in full respect of the Ethical Principles for Medical Research Involving Human Subjects, as adopted by the 18th World Medical Association General Assembly (WMA GA), Helsinki, Finland, June 1964, and subsequently amended by the 64th WMA GA, Fortaleza, Brazil, October 2013. It was approved by the local ethics committees (Board of the Sant'Andrea Hospital, Rome and San Pietro Fatebenefratelli Hospital, Rome).

**Table 1:** Demographic data of both samples (SS, *N* = 289; CS, *N* = 60).

	**Number (*N*)**	**Percent (%)**
**SCREENING SAMPLE (SS)**		
**Nationality**		
Italian	265	91.7%
Non-Italian	24	8.3%
**Marital status**		
Single	4	1.4%
In a relationship	41	14.2%
Married/Cohabiting	238	82.4%
Separated/Divorced	3	1.0%
N/A^a^	3	1.0%
**Education**		
Middle school	13	4.5%
Secondary school/Professional diploma	117	40.%
Graduate/Postgraduate	157	54.3%
N/A^a^	2	0.7%
**Occupation**		
Student	2	0.7%
Unemployed	60	20.8%
Worker	7	2.4%
Employee	149	51.6%
Freelancer	47	16.3%
Manager/Executive position	2	0.7%
Precarious worker	4	1.4%
Other	16	5.5%
N/A^a^	2	0.7%
**Clinical Sample (CS)**		
**Nationality**		
Italian	52	86.7%
Non-Italian	8	13.3%
**Marital status**		
Single	2	3.3%
In a relationship	5	8.3%
Married/Cohabiting	51	85.0%
Separated/Divorced	1	1.7%
N/A^a^	1	1.7%
**Education**		
Primary school	1	1.7%
Middle school	4	6.7%
Secondary school/Professional diploma	24	40.0%
Graduate/Postgraduate	30	50.0%
N/A^a^	1	1.7%
**Occupation**		
Unemployed	14	23.3%
Employed	45	75.0%
N/A^a^	1	1.7%
**Medical conditions**		
No	35	58.3%
Yes	23	38.3%
N/A^a^	2	3.3%
**Current drug treatment**		
No	45	75.0%
Yes	13	21.7%
N/A^a^	2	3.3%
**Psychiatric history**		
No	16	26.7%
Yes	42	70.0%
N/A^a^	2	3.3%
**Previous psychopharmacological therapy**		
No	23	38.3%
Yes	33	55.0%
N/A^a^	4	6.7%
**Current psychopharmacological therapy**		
No	26	43.3%
Yes	34	56.7%
**Psychiatric family history**		
No	19	31.7%
Yes	38	63.3%
N/A^a^	3	5.0%
**Others completed pregnancies**		
No	19	31.7%
Yes	40	66.7%
N/A^a^	1	1.7%
**Abortions**		
No	44	73.3%
Yes	14	23.3%
N/A^a^	2	3.3%
**Past assisted fertilization**		
No	45	75.0%
Yes	11	18.3%
N/A^a^	4	6.7%
**Psychiatric disorders (before, during pregnancy or post-partum)**		
No	23	38.3%
Yes	17	28.3%
N/A^a^	20	33.4%
**Pregnancy complications**		
No	31	51.7%
Yes	25	41.7%
N/A^a^	4	6.7%
**Hospitalizations during pregnancy**		
No	45	75.0%
Yes	11	18.3%
N/A^a^	4	6.6%
**Rest period**		
No	40	66.7%
Yes	17	28.3%
N/A^a^	3	5.0%
**Stressful events in the last years**		
No	17	28.3%
Yes	41	68.3%
N/A^a^	2	3.3%

*Categorical variables*.

a *N/A, data not available. CS, clinical sample; SS, screening sample*.

### Screening Sample

The screening sample included Italian-fluent adult women of the general population screened once during their third trimester of pregnancy (T0) and again 6 months postpartum (T1). We recruited all consecutive women attending fetal monitoring at the Gynecology and Obstetrics unit of San Pietro Fatebenefratelli Hospital of Rome between July and December 2019 during their routine third trimester screening.

Exclusion criteria were failure to provide free informed consent and incomplete comprehension of the Italian language that prevented participants from completing the questionnaires. Participants with an incomplete PASS were also excluded from the final analysis.

Antenatal participants who had consented to being contacted in the postnatal period, were phoned by two trained psychologists of our Center for Prevention and Treatment of Women's Mental Health, 6 months following the birth of their baby and invited to complete the questionnaires again through an online system (Google Form).

### Clinical Sample

CS included antenatal and postnatal psychiatric outpatients referring to the Center for Prevention and Treatment of Women's Mental Health at Sant'Andrea Hospital of Rome (Supplementary Table 1).

### Procedure

Screening tools were administered by physicians and psychologists of our Center for Prevention and Treatment of Women's Mental Health Problems at Sant'Andrea Hospital, Rome, Italy.

Both groups were evaluated using the sociodemographic, clinical and obstetric data collection sheet (Perinatal Interview; PI), the Perinatal Anxiety Screening Scale (PASS), the Edinburgh Postnatal Depression Scale (EPDS), the Temperament Evaluation of the Memphis, Pisa, Paris and San Diego-Autoquestionnaire (TEMPS-A), the Hypomania CheckList-32 (HCL-32), the Zung Self-Rating Anxiety Scale (SAS), and the Mood Disorders Questionnaire (MDQ).

CS patients were also evaluated with The Hamilton Rating Scale for Anxiety [HAM-A; (39)], a semi-structured interview. To assess anxiety diagnosis among patients in the CS group we used the Structured Clinical Interview for DSM-5 (SCID-5).

### Measures

Measures, valid for the purpose of screening symptoms of depression and anxiety, took 30–50 min to complete. Included measures were the following:

**The Perinatal Anxiety Screening Scale** (PASS; 37) is a 31-item self-rated questionnaire investigating anxiety symptoms during the last month in child-bearing women. Each item is rated on a Likert 0–3 scale; the total score is the addition of scores on each item, with higher scores representing more anxiety. Scores may range 0–93. Cutoff for clinical anxiety is ≥26. A further study stratified the investigated population into minimal anxiety (scoring 0–20), mild–moderate anxiety (21–41), and severe anxiety symptoms (42–93) (38). The scale showed adequate test–retest reliability (*rho* = 0.74), a sensitivity of 70% and specificity of 30% at the 26 cutoff. The PASS showed adequate convergent validity, with its global scores significantly correlating with the anxiety subscale of the EPDS and the total EPDS scores, and with both STAI-State and STAI-Trait scores; its reliability is rated excellent for the entire scale (Cronbach's α = 0.96). Its best fit is a four-factor structure explaining 59.37% of total variance. Factor 1 (acute anxiety and adjustment) consists of 8 items related to panic, dissociation and adjustment, factor 2 (general worry and specific fears) includes 10 items related to generalized anxiety (GAD) and phobia, factor 3 (perfectionism, control and trauma) consists of eight items related to obsessive-compulsive (OCD) and posttraumatic symptoms, and factor 4 (social anxiety) is composed of five items focusing on social anxiety (37).

**The Edinburgh Postnatal Depression Scale** [EPDS; (40)] is a 10-item self-report questionnaire administered to screen for depressive symptoms over the past week. The EPDS has been shown to have high reliability and adequate sensitivity and specificity for detecting depressive symptoms in both the antenatal and postnatal periods (41, 42). A score of 10 or greater suggests possible depression. Scores above 12 are likely depressive illness of varying severity. We used the recommended score of 13 or more that indicate probable major depression in postnatal Italian-speaking women (43). Three items of the EPDS (EPDS 3-A, items 3, 4, and 5) have been found to cluster together on an anxiety factor in postpartum women with optimum cut-off scores ranging from 4 to 6 in different studies (44–47). However, the authors maintain that the scale does not confirm an anxiety disorder and does not distinguish whether anxiety scores on these three items are a feature of depression or a distinct entity.

**The Temperament Evaluation of the Memphis, Pisa, Paris and San Diego-Autoquestionnaire** [TEMPS-A; (48)], is a 110-item yes-or-no self-report questionnaire designed to assess affective temperament in psychiatric and healthy subjects. It consists of five temperament-traits, i.e., depressive (D), cyclothymic (C), hyperthymic (H), irritable (I), and anxious (A) subscales. The prevailing temperament is considered the one on which the completer obtains the higher score. We used the validated Italian version (49). The instrument has shown intrasubject diachronic stability (50, 51).

**The Hypomania CheckLList-32** (52) is a self-rating questionnaire investigating lifetime history of hypomanic symptoms. It may be applied to normal people and outpatients. However, it was mainly developed for screening patients with a diagnosis of depression (major depressive disorder, dysthymia, “minor” depression, and recurrent brief depression) for hypomanic symptoms. The total score is the number of positive answers to the 32 items listed in question 3 of the second revision of the tool (HCL-32-R2) (53). Individuals scoring ≥14 are potentially with bipolar disorder/diathesis and should be carefully interviewed. The R2 version, which we used in this study, showed good psychometric properties (54).

**The Zung Self-Rating Anxiety Scale** [SAS; (55)] is a 20-item self-report assessment tool built to measure state anxiety levels, based on scoring in four groups of manifestations: cognitive, autonomic, motor and central nervous system symptoms. Raw scores range from 20 to 80; scores between 20 and 44 are considered to be in the normal range, between 45 and 59 indicate mild-to-moderate anxiety, 60–74 indicate marked-to-severe anxiety, and ≥75 extreme anxiety. The initial cutoff was 50 (56), but the author later lowered it to 45 (57). The best cutoff was later proposed to be 40 for clinical settings and 36 for screening purposes (58). The instrument is suited to investigate anxiety disorders (59) and showed strong correlations with other similar instruments (60).

**The Mood Disorder Questionnaire** [MDQ; (61)] is a 13-item self-report, validated questionnaire (62) designed to assess bipolar diathesis in psychiatric and healthy subjects. Its specificity was found to be 97% and its sensitivity 70% for bipolar type I disorder, much less for bipolar type II disorder (52%) and even less (31%) for bipolar disorder not otherwise specified (63). It is able to detect bipolar propensity (64). The test has shown convergent validity (65) and the same sensitivity as the HCL-32, but better specificity (66).

**The Hamilton Rating Scale for Anxiety** [HAM-A; (39)] was one of the first clinician report rating scales developed to measure the severity of both psychic and somatic anxiety symptoms, and is still widely used today in both clinical and research settings. The scale consists of 14 items, with a total score of 17 or less indicating mild anxiety severity, 18–24 mild-to-moderate, 25–30 moderate-to-severe, and more than 30 severe anxiety.

### PASS Translation

The Italian translation of the PASS was carried out through a direct and reverse translation process (67). Specifically, a bilingual Italian/English psychologist translated the PASS from English to Italian. After that, a bilingual Italian/English researcher back translated the scale.

After discussing any differences between the two translations with the author of the original version of the scale (Dr. Somerville), the scale was translated again into English by a native speaker researcher, unaware of previous translation processes. The Italian version of the PASS includes 31 items. Items are rated as in the original version.

### Data Analyses

Confirmatory Factor Analysis (CFA) was conducted using JASP Version 0.13.1 (68). The quality of the measurement model was examined through the fit indices estimates of Comparative Fit Index (CFI), and Root Mean Square Error of Approximation (RMSEA). According to literature (69), a model is considered to have a good fit if the comparative fit index (CFI) are all >0.90; and the root mean square error of approximation (RMSEA) values are approximately 0.06.

Subsequently, the factor structure of the Italian version of the PASS was analyzed using the Principal Component Analysis (PCA) with oblique (Oblimin) rotation, as recommended (70). To enter in a factor, an item should load with a score of ≥0.4. Should an item score ≥0.4 on more than one factor, the item was assigned to the factor where it obtained the higher score.

The convergent and discriminant validity of the Italian version of the PASS has been assessed by conducting Pearson's correlations between the PASS global score and the EPDS, EPDS 3-A, TEMPS-A, HCL-32 and SAS. Point-biserial correlations were compute between PASS global score and MDQ. The cutoff for all correlations was set at *p* < 0.05.

Test-retest reliability of the PASS was assessed by examining the correlation between the total PASS score in the antenatal and postnatal period for a subsample of participants (*N* = 49) who completed the PASS both antenatally and postnatally.

To assess the diagnostic accuracy of the PASS and to determine the best cutoff score that optimally detected cases defined by a presence or absence of an DSM-5 diagnosis for an anxiety disorder the receiver operating characteristic (ROC) curve analysis was run for a subsample of 60 with an anxiety disorder diagnosis. The IBM SPSS-25 statistical package (IBM Inc., Armonk, New York, 2017) was used for all these analyses.

## Results

### Screening Sample

SS included 312 Italian-speaking adult women of the general population screened once at T0; those 49 who agreed with follow-up were again tested at T1. Participants with an incomplete PASS were excluded from the final analysis (*N* = 23), hence, the final sample consisted of 289 women (*M*_age_ = 33.17 years; *SD*_age_ = 5.08, range, 19–46). All women had currently a partner (*M*_age_ = 36.27 years, *SD*_age_ = 5.64, range, 21–54).

### Clinical Sample

CS included 60 (*M*_age_ = 35.71 years; *SD*_age_ = 5.02, range, 22–50) antenatal and postnatal psychiatric outpatients. CS was composed by 23.3% of women with anxiety disorder (*N* = 14), 13.3% with major depressive disorder (*N* = 8), 30% with comorbid anxiety and depressive disorders (*N* = 18), and 33.4% with other psychiatric diagnoses (e.g., psychosis) (*N* = 20).

Scores obtained on the administered rating scales are shown in Supplementary Table 2. For the MDQ, 22 participants showed a positive result, 190 a negative one.

### Factor Structure of the PASS

CFA revealed inadequate fit indexes for the tested four-factors model proposed by Somerville et al. (37) [*x*2 (428) = 1283.267, *p* < 0.001, CFI = 0.780, RMSEA = 0.085].

Inter-item correlations were sufficiently large for PCA (Bartlett's test of sphericity= χ2 (465) = 4164.67, *p* < 0.001). Sampling adequacy was excellent (Kaiser-Meyer-Olkin test, KMO = 0.92). Four factors were retained based on the results of the scree test (71), the Parallel Analysis test and MAP test (72), which cumulatively explained 52.53% of total variance.

An examination of the factor loadings after Oblimin rotation (Table 2) suggested a slightly different factor structure of the PASS compared to the previous identified (37). More in detail, Factor 1 (Anxiety and worry) had 15 items that addressed symptoms of anxiety, dissociative disorder and adjustment difficulties; Factor 2 (Social anxiety) included six items that addressed social anxiety and adjustment difficulties; Factor 3 (Perfectionism and control) had six items that addressed symptoms of OCD; and Factor 4 (Fears) included four items that addressed GAD and specific fears.

**Table 2:** Factor structure of the Perinatal Anxiety Screening Scale (PASS).

**Scale/item loading factor**	**1**	**2**	**3**	**4**
Factor 1 **Anxiety and worry**				
1. Repetitive thoughts that are difficult to stop or control	0.784			
2. Feeling agitated	0.764			
3. Feeling overwhelmed	0.740			
4. Feeling panicky	0.710			
5. Concerns about repeated thoughts	0.700			
6. Racing thoughts making it hard to concentrate	0.697			
7. Anxiety getting in the way of being able to do things	0.696			
8. Upset about repeated memories, dreams or nightmares	0.680			
9. Fear of losing control	0.669			
10. Feeling detached like you're watching yourself in a movie	0.612			
11. Difficulty adjusting to recent changes	0.612			
12. Worry about the future	0.594			
13. Worry about many things	0.590			
14. Feeling jumpy or easily startled	0.579			
15. Sudden rushes of extreme fear or discomfort	0.375			
Factor 2 **Social Anxiety**				
16. Feeling really uneasy in crowds		0.783		
17. Fear that others will judge me negatively		0.726		
18. Avoiding social activities because I might be nervous		0.701		
19. Worry that I will embarrass myself in front of others		0.684		
20. Avoiding things which concern me		0.418		
21. Losing track of time and can't remember what happened		0.303		
Factor 3 **Perfectionism and Control**				
22. Wanting things to be perfect			0.815	
23. Needing to be in control of things			0.814	
24. Having to do things in a certain way or order			0.806	
25. Difficulty stopping checking or doing things over and over			0.553	
26. Being “on guard” or needing to watch out for things			0.433	
27. Difficulty sleeping even when I have the chance to sleep			0.334	
Factor 4 **Fears**				
28. Fear that harm will come to the baby				0.856
29. Worry about the baby/pregnancy				0.844
30. A sense of dread that something bad is going to happen				0.641
31. Really strong fears about things, e.g., needles, blood, birth, pain, etc.				0.519
**Cronbach's alpha**	0.926	0.721	0.816	0.781
**% of variance explained**	34.68	6.56	6.40	4.89

Three items with factor loading below the 0.4 threshold were retained due to their clinical relevance. These items were the following: item 15 “Sudden rushes of extreme fear or discomfort,” item 21 “Losing track of time and can't remember what happened,” and item 27 “Difficulty sleeping even when I have the chance to sleep.” There was only one cross-loading item, i.e., item 23, “Avoiding things which concern me” and was retained in the factor showing the highest loading and consistency with clinical anxiety presentations, i.e., Factor 2 (Social anxiety). The four subscales had high reliabilities (Cronbach's α ranged from 0.721 to 0.926) and were weakly to moderately correlated (*r* values ranged from 0.16 to 0.46). The entire scale showed also excellent reliability (Cronbach's α = 0.929).

### Construct Validity of the PASS

The PASS global score was significantly correlated with all the scales employed, except for the TEMPS-A and the MDQ, which are indicative of adequate convergent and discriminant validity (Table 3).

**Table 3:** Correlations matrix between the PASS total score and other scales.

			**TEMPS**									
		**PASS**	**Depressive**	**Cyclothymic**	**Hyperthymic**	**Irritable**	**Anxious**	**HCL-32**	**EPDS 3-A**	**EPDS**	**SAS**	MDQ^*^
PASS	*r*	-	0.532	0.592	−0.111	0.541	0.702	0.280	0.618	0.735	0.542	0.121
	*p*		**<0.001**	**<0.001**	0.079	**<0.001**	**<0.001**	**<0.001**	**<0.001**	**<0.001**	**<0.001**	0.078
	*N*	289	256	255	252	250	250	229	285	288	280	212

* *Correlation between the PASS total score and the MDQ was a point-biserial correlation (r_pb_)*.

*EPDS, Edinburgh Postnatal Depression Scale; EPDS 3-A, Edinburgh Postnatal Depression Scale, Anxiety items; HCL-32, Hypomania CheckList-32; MDQ, Mood Disorder Questionnaire; PASS, Perinatal Anxiety Screening Scale; SAS, Zung Self-rating Anxiety Scale; TEMPS-A, Temperament Evaluation of Memphis, Pisa, Paris and San Diego-Autoquestionnaire*.

*All significant values in bold*.

### Test-Retest Reliability

The Pearson correlation coefficients were calculated to assess the test-retest reliability of the PASS for a subsample of participants (*N* = 49) who completed the PASS antenatally and postnatally (with a 6-month interval). The correlation for the PASS global scores was 0.482, *p* < 0.001.

### Screening Accuracy of the PASS

**Figure 1:**
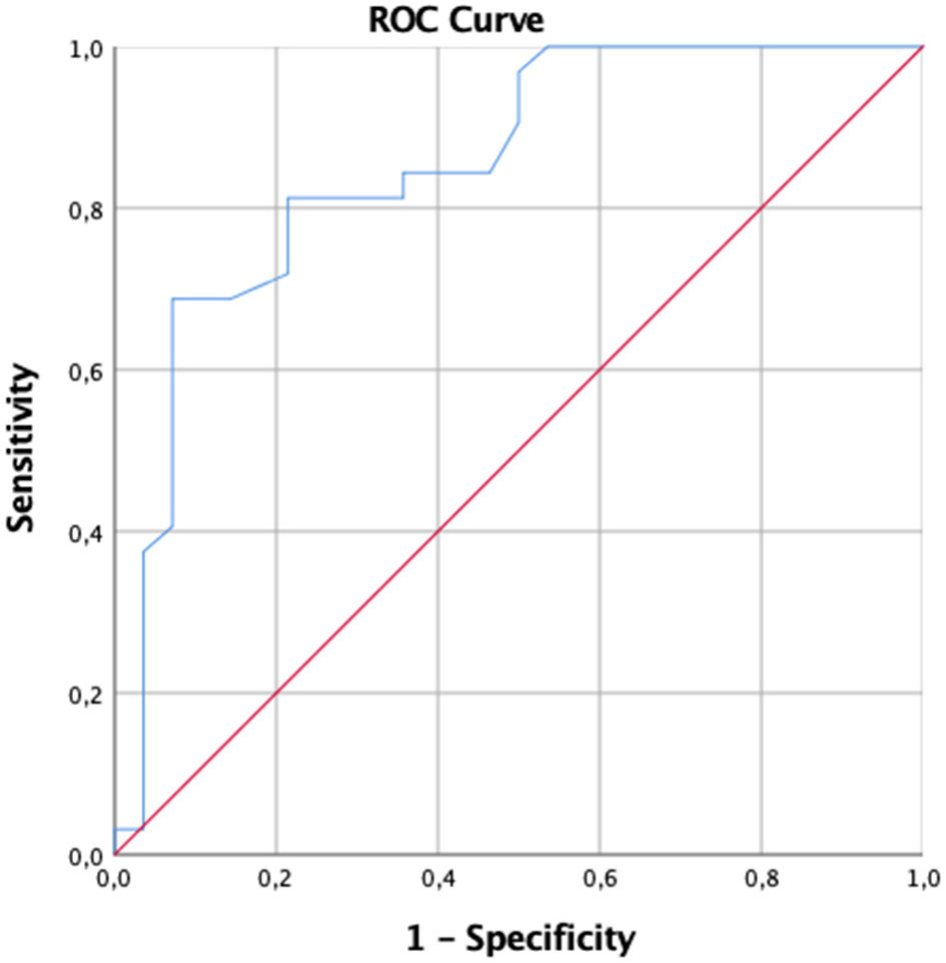
Graphical representation of receiver operator characteristic curve of the PASS total score.

In line with the hypothesis, the PASS offers a good diagnostic accuracy within the collected clinical sample. PASS total score showed an AUC value of 0.852 (*SE* = 0.051) (Figure 1). Additionally, because in a clinical assessment, sensitivity has priority over specificity, given the high cost of false-negative errors, we also identified the PASS cutoff that would yield, in our data, values of sensitivity as close as possible to 90. This value is 25.5 with a positive predictive power (PPP) of 69% and a negative predictive power (NPP) of 93%. For the screening sample, these figures were 77.94 and 86.96%, respectively. The results revealed that the PASS, at the recommended cutoff score of 26 detected correctly 96.9% of women with anxiety disorder, performing better than the EPDS 3-A, SAS and HAM-A. Indeed, EPDS 3-A, with its cutoff of 6, detected 86.7% (AUC = 0.799) of the cases; SAS, with a cutoff of 45 detected 42.9% (AUC = 0.693) of the cases, whereas the HAM-A at a cutoff of 18 detected 88.9% (AUC = 0.714) (Supplementary Table 3). A significant and positive correlation was found between the PASS and the HAM-A (*r* = 0.577).

## Discussion

In this study, we showed the validity and reliability of the Italian version of the PASS in assessing anxiety symptoms and disorders in general and clinical perinatal samples and a better diagnostic accuracy than other valid instruments. Factor analysis showed a four-factor structure with PCA, which however differed from the original version, as assessed with CFA. The PASS showed construct validity and convergence with other reliable measures and test-retest reliability; it also showed excellent sensitivity and specificity, as shown by an AUC of 0.85 at the same cutoff of 26, as per the original version. This is above the 0.8 cutoff suggested by Fairbrother et al. (73), it should be noted that in this study, only one, non-specific scale was found to perform above this cutoff, while both EPDS and EPDS 3-A were found to have an AUC of 0.744 and 0.757, respectively.

It is important to assess anxiety symptoms and disorders in perinatal women through valid and reliable measures (32, 74). A careful assessment may provide important information for the medical staff and for women themselves on the challenges and burden experienced with pregnancy and maternity. If perinatal anxiety is detected, women's well-being and motherhood could improve by providing prompt psychological support. From this perspective, we evaluated the psychometric properties of the PASS Italian version in a sample of women undergoing mental health screening during their third trimester of pregnancy. Overall, findings confirm that the Italian version of the PASS is a reliable and valid questionnaire for assessing anxiety symptoms in the perinatal period.

Consistently with the original model (37), the PCA has identified a four-factor structure, slightly different from the previous one in item composition. Slightly different item compositions of the four identified factors were also observed with the Turkish (75), Bangladeshi (76), Iranian (77), and Sri-Lankan versions of the PASS (78), while the Arabic versions diverged, with the Saudi Arabian one agreeing on the four-factor solution (79), while the Lebanese found seven (80) (submitted). However, this is a frequent encounter in literature, especially when some years elapse from one factor analysis to another [*cfr*. (81) *vs*, (82)]. The reliability of the scores of the Italian version of the PASS, assessed trough their internal consistency, was excellent or at least good, as indicated by Cronbach's alpha coefficients ranging from 0.72 to 0.93.

Our results supported the convergent validity of the Italian version of the PASS through significant and positive correlation with other measures of anxiety, showing higher correlation values with the EPDS, TEMPS-A and the EPDS 3-A. The PASS total score correlated significantly and positively also with measures of depression. Discriminant validity of the PASS was substantiated by the lack of significant correlations with the MDQ and the TEMPS-A.

Another interesting result emerges from test-retest reliability. Compared to the retest correlation coefficient (*r* = 0.74) reported by Somerville et al. (37), we found a lower association between the T0 and T1. This lower association with respect to Somerville et al. (37) could be attributed to the antenatal-postnatal interval; in fact, in that study patients were contacted 2–6 months after delivery, while we contacted our subsample 6 months after delivery. The possibly longer interval could account for this slight difference, as anxiety related to childbirth tends to progressively subside after delivery (8).

The PASS had similar sensitivities and specificities in both the SS and the CS, as shown by PPP and NPP; this means that it is suitable for both general screening and clinical application in women with clinically significant anxiety.

Aligning with the validation study (37), the screening accuracy indicated that the Italian PASS performed better in detecting women with anxiety disorder diagnosis than the other measures employed, which are frequently used in clinical practice. It is interesting to notice that we found a positive correlation between the PASS, a self-report measure, and the HAM-A, a clinician-rated instrument. This correlation, together with the higher classification accuracy of the PASS, could support the use of the PASS in clinical settings where saving time and resources for the practitioners are mandatory. Giving higher priority to sensitivity over specificity, we identified an optimal threshold of 26, the same found by Somerville et al. (37). At this cutoff score, 97% of women with an anxiety disorder were detected and, consequently, may benefit from immediate psychological assessment and support. Furthermore, it is worth noting that about 18% of the SS (*N* = 52 of 289) score above the cutoff of 26, in line with epidemiological data on the prevalence of anxiety disorder in women during the perinatal period.

### Limitations and Strengths

The main limitation of this study is the small sample size that did not allow for investigating differences among its subgroups.

It is commonly believed that self-rating is inferior to clinician-rating in validity (83); however, this by no means the rule (84), and the combined use of both types of tools has been advocated to rate anxiety (85, 86). We here used the HAM-A to cross-validate the PASS, and the latter outperformed the clinician-rated tool in the CS.

The implications for future research would entail looking at the interventions needed to further decrease the effects of maternal pregnancy anxiety both for parenting style and child's development (87).

The PASS is the first screening and clinical tool available in Italian to assess perinatal anxiety. The PASS is designed for use in heterogeneous populations and could be easily administered by obstetrics staff in everyday clinical practice. A simple screening task completed once per trimester during pregnancy could help clinicians to target women who are most in need of mental support. Furthermore, women who are experiencing pregnancy-specific anxiety can recognize themselves over the items of the PASS.

We strengthened the validation of the Italian version of the PASS using a standardized diagnostic interview, the HAM-A in a clinical population and obtained a good convergence.

Due to our design, we did not assess the validity of the instrument in the same woman across the entire prepartum and postpartum periods, so we have to limit our conclusions to late-pregnancy and 6 months postpartum. Future studies will confirm and extend the validity to the entire perinatal period.

## Conclusions

Assessing anxiety in prepartum and postpartum with specific scales is crucial for new mothers' mental health and new-born babies' upbringing. The PASS showed to be reliable to this end more than other existing instruments. We suggest that its use should enter standard clinical practice.

## Data Availability Statement

The datasets presented for this article are available and contained in the online Supplement. Further requests regarding datasets should be directed to giorgio.kotzalidis@uniroma1.it.

## Ethics Statement

The studies involving human participants were reviewed and approved by Board of the Sant'Andrea Hospital, Rome and San Pietro Fatebenefratelli Hospital, Rome. The patients/participants provided their written informed consent to participate in this study.

## Author Contributions

AK, LD, and GAn conceived and designed the study. CM, LD, GK, and AS performed statistics. AS, GK, GAr, and GC performed literature searches. AK, LD, GAn, GAr, GC, and MB saw the patients involved in the study. GAn and MB addressed ethical issues. GK, GAr, LD, AK, and GAn translated the scale. AK, LD, CM, and GAn wrote the first draft. AK, LD, GS, AS, GP, and GK wrote the Introduction. CM, LD, GK, GAr, GP, and GC wrote the Methods and Results. SF, AK, LD, PR, SS, GP, GK, GAr, and GC wrote the Discussion. SS, AK, LD, GK, SF, GS, AS, and GAn supervised the final draft. All authors viewed and approved the final version.

## Conflict of Interest

The authors declare that the research was conducted in the absence of any commercial or financial relationships that could be construed as a potential conflict of interest.
