# Supplementary material for: Psychometric Properties of the Perinatal Anxiety Screening Scale Administered to Italian Women in the Perinatal Period
Source: Front Psychiatry. 2021 Jun 22;12:684579. doi: 10.3389/fpsyt.2021.684579 (PMC8257936; doi:10.3389/fpsyt.2021.684579)
Supplement: Supplementary file 1 [file Data_Sheet_1.PDF]

Supplement for

Psychometric properties of the Perinatal Anxiety Screening Scale (PASS) administered to Italian women in the perinatal period

Alexia **Koukopoulos**,*[1,2] Cristina **Mazza**,[3] Lavinia **De Chiara**,*[2,4] Gabriele **Sani**,[5,6] Alessio **Simonetti**,[1,2,7] Georgios **D. Kotzalidis**,^[2,4] Giulia **Armani**,[4,8] Gemma **Callovini**,[2,9] Marco **Bonito**,[10] Giovanna **Parmigiani**,[4] Stefano **Ferracuti**,[1] Susanne.**Somerville**,[11] Paolo **Roma**,[1] Gloria **Angeletti**[2,4]

[1] Department of Human Neuroscience, Sapienza University of Rome, Rome, Italy

[2] Lucio Bini Centre, Rome, Italy

[3] Department of Neuroscience, Imaging and Clinical Sciences G. d'Annunzio University of Chieti-Pescara

[4] Department of Neurosciences, Mental Health, and Sensory Organs (NESMOS), Sapienza University of Rome, Faculty of Medicine and Psychology, Sant'Andrea University Hospital, Rome, Italy

[5] Institute of Psychiatry, Università Cattolica del Sacro Cuore, Rome, Italy

[6] Department of Psychiatry, Fondazione Policlinico Universitario "Agostino Gemelli" IRCCS, Rome, Italy

[7] Menninger Department of Psychiatry and Behavioral Sciences, Baylor College of Medicine, Houston, TX, United States

[8] APC Associazione di Psicologia Cognitiva, Rome, Italy

[9] Department of Mental Health, Psychiatric Service of Diagnosis and Treatment, "San Camillo de Lellis" NHS Hospital, ASL Rieti, Italy

[10]Dipartimento Materno Infantile, San Pietro Fatebenefratelli Hospital, Rome, Italy

[11]Department of Psychological Medicine, King Edward Memorial Hospital, Australia

*These authors equally contributed to the writing of the manuscript.

^ Correspondence: Georgios D. Kotzalidis, MD, PhD; Department of Neurosciences, Mental Health, and Sensory Functions (NESMOS), Sapienza University of Rome, Faculty of Medicine and Psychology, Sant'Andrea University Hospital, Via di Grottarossa 1935-1939, 00189 Rome, Italy. Tel. +39-0633775951; Fax: +39-0633775342; e-mail: giorgio.kotzalidis@uniroma1.it

Author E-mails: alexia.koukopoulos@uniroma1.it lavinia.dechiara@uniroma1.it cristina.mazza@unich.it gabriele.sani@unicatt.it alessio.simonetti@bcm.edu giorgio.kotzalidis@uniroma1.it giuliaarmanipsi@gmail.com g.callovini@asl.rieti.it bonitomarco@libero.it giovanna.parmigiani@uniroma1,it stefano.ferracuti@uniroma1.it Susanne.Somerville@health.wa.gov.au paolo.roma@uniroma1.it gloria.angeletti@uniroma1.it

Table S1. Clinical Sample composition according to timing of assessment.

	**Frequency**	**%**	**PASS *M* (SD)**
I Trimester Pre	8	13.3	37.25 (19.8)
II Trimester Pre	5	8.3	46.40 (24.8)
III Trimester Pre	7	11.7	35.43 (17.4)
1 Month Post	5	8.3	31.20 (12.2)
2-3 Months Post	9	15	43.67 (21.9)
4-6 Months Post	13	21.7	37.92 (27.2)
7-12 Months Post	13	21.7	41 (16.7)
Total	60	100	39.22 (20.41)

Abbreviations: M, mean; PASS, Perinatal Anxiety Screening Scale; Pre, prepartum; Post, postpartum; SD, standard deviation

Table S2. Scores on psychometric self-rating scales in the Screening Sample (SS) (continuous variables).

**Scale**	**N**	**Mean**	**SD**
PASS	289	15.52	12.4
EPDS	288	5.94	4.6
TEMPS-A Depressive	256	5.88	2.8
TEMPS-A Cyclothymic	255	3.38	3.1
TEMPS-A Hyperthymic	252	10.75	3.9
TEMPS-A Irritable	250	2.12	2.4
TEMPS-A Anxious	250	5.58	4.5
HCL-32	229	12.08	6.7
SAS	280	34	6.9

Abbreviations: EPDS, Edinburgh Postnatal Depression Scale; HCL-32, Hypomania CheckList-32; PASS, Perinatal Anxiety Screening Scale; SAS, Zung Self-rating Anxiety Scale; SD, standard deviation; TEMPS-A, Temperament Evaluation of Memphis, Pisa, Paris and San Diego-Autoquestionnaire

Table S3. Scores on PASS, EPDS-A, SAS, and HAM-A in the Clinical Sample (CS)

**Scales**	***Mean (SD)***	***Min-Max***
PASS	39.22 (20.41)	1-85
EPDS-A	5.77 (2.5)	0-9
SAS	39.77 (8.3)	27-65
HAM-A	13.62 (8.7)	0-35

Abbreviations: EPDS-A, Edinburgh Postnatal Depression Scale, anxiety items; HAM-A, Hamilton Anxiety Rating Scale; *M*, mean; PASS, Perinatal Anxiety Screening Scale; SAS, Zung Self-rating Anxiety Scale; SD, standard deviation
